# Characterization of Drought-Responsive Transcriptome During Seed Germination in Adzuki Bean (*Vigna angularis* L.) by PacBio SMRT and Illumina Sequencing

**DOI:** 10.3389/fgene.2020.00996

**Published:** 2020-08-31

**Authors:** Zhenzhen Zhu, Hongwei Chen, Ke Xie, Changyan Liu, Li Li, Liangjun Liu, Xuesong Han, Chunhai Jiao, Zhenghuang Wan, Aihua Sha

**Affiliations:** ^1^Hubei Collaborative Innovation Center for Grain Industry/Engineering Research Center of Ecology and Agricultural Use of Wetland of Ministry of Education, Yangtze University, Jingzhou, China; ^2^Institute of Food Crops, Hubei Academy of Agricultural Sciences/Hubei Key Laboratory of Food Crop Germplasm and Genetic, Wuhan, China; ^3^Zhongzhi International Institute of Agricultural Biosciences, Biology and Agriculture Research Center, University of Science and Technology Beijing, Beijing, China

**Keywords:** adzuki bean, drought, seed germination, SMRT, RNA-seq

## Abstract

The full-length single-molecular sequencing and short reads Illumina sequencing were combined to generate the transcripts of adzuki bean with high-quality. A total of 17,636 loci and 60,454 transcripts were detected in this study. To characterize the drought-responsive genes during seed germination in adzuki bean, two varieties, i.e., tolerant and sensitive to drought stress, were selected to conduct analysis of alternative splicing dynamics (AS) and differentially expressed genes (DEGs) by combining the newly assembled draft genome and public adzuki bean reference genome. AS analysis indicated that both the two varieties underwent a little more AS events under control conditions than under drought stress. Among the AS events, IR (intron retention) predominately accounted for 34.3%, whereas AD (alternative donor site) was the least frequent with 15.8%. Meanwhile, 562 long non-coding RNAs, 409 fusion genes and 1208 transcription factors were identified. Moreover, a total of 5,337 DEGs were identified in comparison of the two varieties with drought or control treatments. Notably, 82 DEGs were discovered in the two varieties under drought stress, which might be the candidate in regulation of seed germination to answer for different drought tolerance. The DEGs encoded proteins involved in primary or second metabolism, plant hormone signal transduction, transcript or translation processes, ubiquitin proteasome system, transcription factor, transporters, and so on. The results facilitate to increase the knowledge about the mechanism of drought tolerance during crop seed germination, and provide reference for the breeding of drought-tolerant adzuki bean.

## Introduction

Adzuki bean (*Vigna angularis* L.) is widely planted in East Asian countries such as China, Japan and Korea as a second-most important crop of legume (Lestari et al., 2014). Seeds of adzuki bean are rich in protein, starch, mineral elements, and vitamins (Xu et al., 2008). Adzuki bean can be used to make desserts or pastry filling (Chen et al., 2015) as well as medicine such as adiuretic and antidote. Therefore, it is not only a nutritious food but also one significant raw material of food and beverage industry for people’s life and health.

Drought stress can decrease crop yield and reduce the water available in the soil (Orsenigo et al., 2017). The severity of damage caused by drought varies among different crops and at different growth stages (Shah et al., 2017). In many crops, seed germination is one critical phase that is greatly influenced by drought in plant life (Ribeiro et al., 2018). Water stress had more sensitive impact on plumule than any other stress (Abdoli and Saeidi, 2012), thus, drought determines the rate of germination and the establishment of seedlings to a great extent. Adzuki bean is always grown in poor-soil regions because it has broad adaption and high tolerance to abiotic stress (Yang et al., 2015). However, seed germination of adzuki bean is sensitive to drought (Ohya et al., 2002). Therefore, it is significant to exploit key genes that are involved in drought response during seed germination of adzuki bean, which will provide potential resource to improve drought tolerance and to increase yield. The completion of genome sequencing paved the way for the genomic and functional studies in adzuki bean (Yang et al., 2015).

Adzuki bean possesses a rather smaller genome size of approximately 500 Mb. The genome sizes of other main legume crops such as soybean, pea, and faba bean were about 1200, 4000, and 13000 Mb, respectively (Cubero, 1974; Pearce, 1996; Chen et al., 2015). Adzuki bean can be used as a model species, especially for non-oil seed legumes due to its short growth period and small genome size (Parida, 1990; Yamada et al., 2001). Recently, a number of SSRs (simple sequence repeats) and SNPs (single-nucleotide polymorphisms) were exploited due to the developmental genomics in adzuki bean (Chen et al., 2015; Kang et al., 2015). The genetic map resolution was also improved based on the SNP and SSR markers (Wang et al., 2012; Suzuki et al., 2013; Lestari et al., 2014). Meanwhile, bZIP genes were demanded to be responsive to drought and salt stress in *Vigna radiata* and *Vigna angularis* with whole-genome sequences and quantitative real-time PCR analysis (Wang et al., 2018). However, the information of genome-wide analysis for drought response is still unavailable in adzuki bean.

The transcriptome can display the expression of all genes in specific cell or tissue (Cheng et al., 2017). RNA sequencing (RNA-Seq) makes it possible to capture these genes (Cheng et al., 2017). In recent years, fundamental changes happened for the sequencing technology (Alghamdi et al., 2018). The second generation sequencing (NGS) technology deeply enhanced the efficiency and speed in gene discovery (Mortazavi et al., 2008; Asmann et al., 2009; Hershkovitz et al., 2013). However, it is not able to predict each isoform accurately due to the short sequencing reads. By contrast, single-molecule real-time (SMRT) sequencing technology, such as the PacBio system, yields sequence reads with kilobase size which are generally considered as full-length mRNA molecules (Cheng et al., 2017; Li et al., 2017; Wang M. et al., 2017). Hence, SMRT were applied in many plant studies and provided further information on transcript diversity such as alternative splicing and alternative polyadenylation (Minoche et al., 2015; Abdel-Ghany et al., 2016; Wang et al., 2016).

In this study, two adzuki bean varieties with contrast drought tolerance were selected for transcriptome analysis by PacBio SMRT sequencing and Illumina sequencing technology. The objective is to investigate genes participating in drought regulation during seed germination. A more complete full-length transcripts were generated by the hybrid sequencing strategy, which was valuable for further genome annotation. Different AS events and a number of DEGs were identified between the two varieties under drought stress during seed germination, implying that they were crucial for regulate seed germination upon drought in adzuki bean. The findings facilitated understanding of the drought tolerance mechanism of crops during seed germination, and provided reference for the breeding of adzuki bean varieties to improve drought tolerant.

## Results

### Transcriptome Profiles of Adzuki Bean During Germination Upon Drought Stress

A total of 269,311,737 paired-end short reads were produced across all libraries by NGS, and 456,416, 374,530 circular consensus sequence reads were yielded for s17033 and s17235 by SMART sequencing, respectively (Supplementary Table S1). The full length and non-chimeric (FLNC) reads were distinguished by the inclusion of 5′ primer, 3′ primer, and 3′ poly(A) tails from the circular consensus sequence reads. A total of 470,296 high-quality FLNC reads were obtained after error correction with Illumina short reads, FLNC reads mapping and a filtration procedure (see section “Materials and Methods”). Among them, 91.83% were uniquely mapped to the annotated adzuki bean genomic version of Vigan 1.1^1^ (Supplementary Table S2).

**FIGURE 1:**
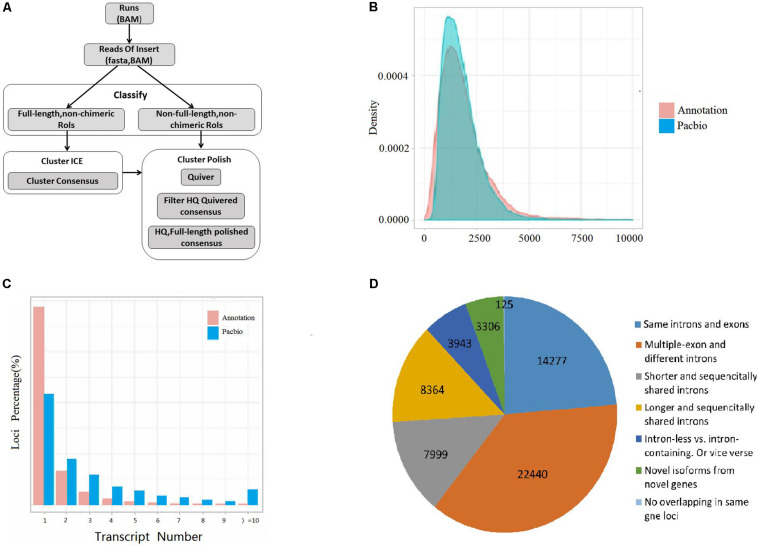
Experimental workflow and comparison of isoform from annotation and PacBio. **(A)** Iso-Seq workflow for data processing (ToFU). **(B)** Comparison of transcript length between the Vigan 1.1 annotation and the PacBio date. **(C)** Comparison of the isoform number between the Vigan 1.1 annotation and the PacBio date. **(D)** Structure comparison of Vigan 1.1 annotation and the PacBio data.

A total of 60,454 non-redundant transcripts were collapsed from the uniquely mapped FLNC reads. The average length was 1,822 bp, which was longer than the length 1,733 bp of the annotated transcripts in public reference genome Vigan 1.1 (Figure 1B and Supplementary Table S3). The 60,454 transcripts were produced from 17,636 gene loci, which covered 63.49% of the annotated genes in Vigan 1.1. 56.91% of the 17,636 gene loci were multiple-exon genes that encode two or more isoforms, whereas the percentage of multiple-exon genes was 22.47% in the public annotated adzuki bean genome (Figure 1C). The results highlighted that the hybrid sequencing strategy significantly improved the length and the quantity of transcripts compared to the annotated adzuki bean genome.

### Characterization of Newly Discovered Loci and Isoforms

The PacBio transcripts were divided into seven groups compared to the Vigan 1.1 annotation (Figure 1D and Supplementary Figure S1). In total, 2,457 (9.67%) novel genes were identified with no any overlap with the annotated genes in the Vigan 1.1. The novel genes encoded 3,306 isoforms with 1804 single-exons and 1502 multiple-exons (Supplementary Table S4). Meanwhile, 42,871 novel isoforms were identified from 10,966 annotated genes in the public reference genome Vigan 1.1 (Supplementary Table S4). These results indicated that the hybrid sequencing strategy generated a set of high-quality transcripts in adzuki bean.

Furthermore, we searched the newly isoforms against the public databases. The results indicated that 91.17% of the novel isoforms corresponding to novel genes and 99.27% of the novel isoforms transcribed by annotated genes showed similarity to the annotated proteins previously (Supplementary Table S5). For the remaining newly discovered isoforms with no sequence similarity to previously annotated proteins, a total of 286 (8.65%) and 276 (0.6%) novel genes and annotated genes derived isoforms were identified as long non-coding RNAs (LncRNAs) by blast against LncRNA database, respectively. It highlighted the advantage of PacBio sequencing in identifying LncRNAs (Abdel-Ghany et al., 2016; Wang et al., 2016) and provided a comprehensive LncRNA database in adzuki bean.

### AS Dynamics in the Germinating Seeds Under Drought Stress or Not

**FIGURE 2:**
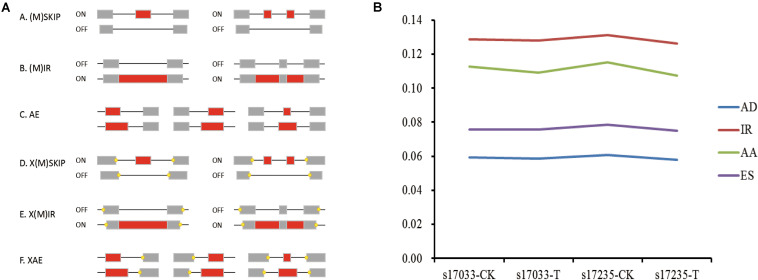
Visualization of alternative splicing (AS) modes and dynamic in four samples. **(A)** Skipping of single exon (SKIP) and multiple exons (MSKIP) (A); Retention of single (IR) and multiple (MIR) introns (B). Alternative exon ends (5′, 3′, or both) (AE) (C). Exon skipping and alternative exon ends simultaneously occurred in one isoform, and the yellow points represent alternative exon ends (D). Intron retention and alternative exon ends simultaneously occurred in one isoform, and the yellow points represent alternative exon ends (E). Multiple exons that had undergone AE in one isoform (F). **(B)** Line chart showing the percentage of each of the four main AS types (Y-axis),namely AA (Alternative Acceptor site), IR (Intron Retention), AD (Alternative Donor site), and ES (Exon Skipping), among the four samples (x-axis).

AS regulates eukaryote trait expression as an important mechanism (Nilsen and Graveley, 2010). AS events were detected in the germinating seeds of s17235 and s17033 under drought stress or not (s17235-T, s17235-CK, s17033-T, s17033-CK) by SMRT sequencing on the PacBio Sequel platform. Four major types of AS were characterized, that is, exon skipping (ES), alternative acceptor site (AA), alternative donor site (AD) and intron retention (IR) (Figure 2A). The combined percentage of the four AS types accounts for roughly 37% of all the AS events among all the samples (Supplementary Table S6). On the whole, control groups have a little bigger amount in each AS types than stress groups (Figure 2B). In addition, IR predominated among four AS events, accounting for 34.3%, whereas AD (15.8%) was the least frequent. Function classification by GO and KEGG indicated that ‘Spliceosome,’ ‘protein processing in endoplasmic reticulum,’ and ‘RNA transport’ were mainly assigned in KEGG pathway, whereas ‘binding processes’ were significantly enriched by GO assignment for genes with differential IR (Supplementary Figure S2 and Supplementary Table S7).

Moreover, a total of 1,397 and 1,356 genes with specific AS event in s17033-T and s17235-T were identified, respectively. Among them, there were 152 differentially spliced genes (DSGs), including one newly discovered genes (NC_030637.1.913) in s17033-T vs. s17235-T (Supplementary Table S8). IR is the primary pattern of AS in DSGs, accounting for 69.93%, whereas ES (3.92%) was the least frequent. As for GO assignments, the dominant terms were ‘metabolic process,’ ‘cellular process,’ and ‘single-organism process’ in the biological process ontology; ‘cell part,’ ‘cell,’ and ‘membrane’ were the principal of the cellular components; ‘catalytic activity’ and ‘binding’ were dominant in molecular function (Supplementary Figure S3). Out of 152 DSGs, 70 were mapped to 32 KEGG pathways, such as ‘biosynthesis of secondary matabolites,’ ‘phenylpropanoid biosynthesis,’ ‘protein processing in endoplasmic reticulum,’ ‘starch and sucrose metabolism,’ and ‘spliceosome’(Supplementary Table S9 and Supplementary Figure S3).

### Identification of Fusion Genes and Transcription Factors

A total of 133 genes corresponding to 409 full-length transcripts were identified as fusion genes, which have more than one locus in the genome (Supplementary Table S10). Among them, 22 chimeric genes (16.54%) corresponding to 299 (73.11%) fusion isoforms were supported at least two FLNC, whereas the remaining were supported only one (Supplementary Table S11). Besides, two fusion categories were identified, that is, the inter-chromosome and intra-chromosome, which means the fusion fragments from different chromosomes or from the same chromosome, respectively. The number of inter-chromosomal fusion genes (121) was greater than intra-chromosomal fusion genes (12).

Transcription factors (TFs) are essential for gene expression regulation, which have been demonstrated to regulate drought tolerance in plants (Fang et al., 2008; Sun et al., 2016; Mehta et al., 2017). A total of 1,208 transcription factors classified into 45 known families were identified. The largest group was AP2-EREBP (190, 15.73%), followed by MYB-related (156, 12.91%), MYB (143, 11.84%), bHLH (80, 6.62%), GRAS (79, 6.54%) (Supplementary Table S12), which were reported to have great effect on regulating drought response in plants (Mehta et al., 2017). NAM-ATAF-CUC2 (NAC) is a plant-specific TF family with a highly conserved DNA-binding domain, and many genes belonging to this family are responsive to drought stress (Fang et al., 2008). There were 57 genes encoding NAC TF family (Supplementary Table S12). Similarly, genes for TF families such as HSF (Li M. Y. et al., 2014; Li P. S. et al., 2014), C2H2 type zinc Finger (Shi et al., 2014), C3H (Mehta et al., 2017), WRKY (Ren et al., 2010), bZIP (Zhong et al., 2015), HB (Jin et al., 2014), zf-HD (Tran et al., 2007) were identified which were reported to play important roles in response to drought stress, and were also highly abundant in our transcriptome dataset.

### Gene Expression Levels in Germinating Seeds of Two Adzuki Bean Varieties Under Drought Stress or Not

To assess changes in gene expression levels in germinating seeds under drought stress or not, Illumina sequencing was compared to the set of transcripts constructed from known transcripts and new transcripts obtained from PacBio sequencing by means of Bowtie2. The expression levels of genes were quantified by RPKM (reads per kilobase per million mapped reads) that were normalized by the mapped reads. A total of 90,702 genes were detected in the two samples, of which, 67,524 (74.45%), 71,303 (78.61%), 67,921 (74.88%), and 71,274 (78.58%) genes were expressed (RPKM > 0) in s17033-24-T, s17033-24-CK, s17235-24-T and s17033-24-CK, respectively. Notably, 43.8% of the genes are highly expressed (RPKM ≥ 1000). The most highly expressed genes encoded defensin-related protein, late embryogenesis abundant protein, and seed maturation-related protein (Supplementary Table S13). Other highly expressed genes included those related to plant stress such as metallothionein-like protein (108328551, 108329743), 18 kDa seed maturation protein (108323470, 108323471), universal stress protein (108325353, 108324161), NAC domain-containing protein (108321636). Intriguingly, some genes associated with adversity stress were exclusively highly expressed in drought-tolerant variety, such as heat shock 70 kDa protein (108320180), temperature-induced lipocalin-1-like (108330587) which were expressed in 17235-T, snakin-2-like (108337129), scarecrow-like protein (108333499), and abscisic acid receptor PYL4 (NC_030643.1.155) which were expressed in 17235-CK (Supplementary Table S13).

To evaluate the reproducibility of RNA-Seq data of the three biological replicates for each samples, the correlation analysis were conducted by the RPKM values of the all 12 samples. The high correlations of gene expression levels among replicates were detected with an average coefficient of 0.9583, 0.9076, 0.8923 and 0.9346 for s17033-24-CK, s17033-24-T, s17235-24-CK, and s17235-24-T, respectively (Supplementary Figure S4). Therefore, it demonstrated a good reproducibility of the gene expression data.

### DEG Identifying and Functional Categorizing

**FIGURE 3:**
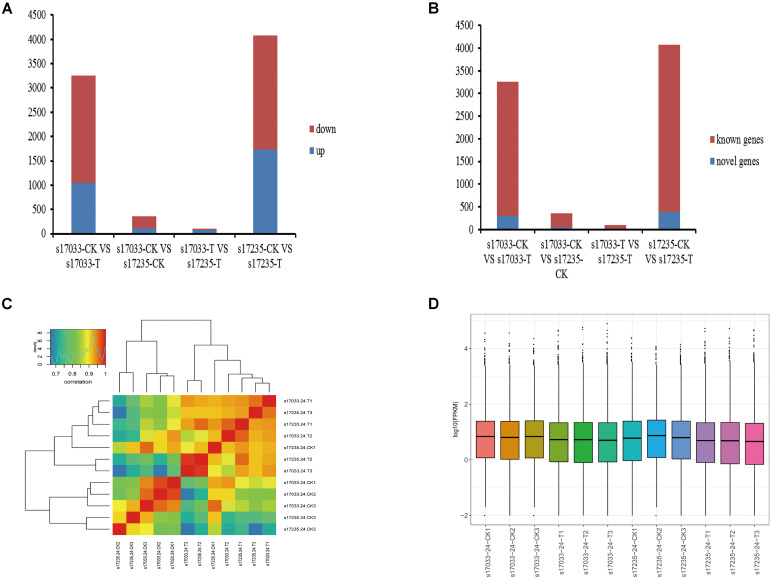
The expression analysis of DEGs. **(A)** Number of up-regulated or down-regulated DEGs by pairwise comparison. Blue, up regulated; Red, down- regulated **(B)** Number of novel or known DEGs by pairwise comparison. Blue, Novel DEGs; Red, Known DEGs. **(C)** The correlation of responsive transcriptomes for each samples. **(D)** The boxplot of FPKM in each sample.

Firstly, we analyzed DEGs (fold change ≥ 2.0, FDR < 0.05) in the comparisons of drought stress with the control in both varieties. There were 3,256 DEGs (31.91% up regulated) and 4,074 DEGs (42.32% up-regulated), including 295 and 388 novel genes, in s17033 and s17235, respectively (Figure 3, Supplementary Figure S5, and Supplementary Table S14). It implied a number of DEGs were induced in response to drought in both varieties. Among them, 2,195 DEGs were identified in both varieties (Supplementary Figure S5 and Supplementary Table S14), inferring that they may play basic defense to drought regardless of variety difference. GO analysis indicated that the 2,195 DEGs were mainly distributed in terms of “metabolic process,” “cellular process,” “catalytic activity,” “binding,” “cell,” and “cell part.” The main KEGG pathways were “global and overview maps,” “carbohydrate metabolism,” and “lipid metabolism” (Supplementary Figure S6).

**FIGURE 4:**
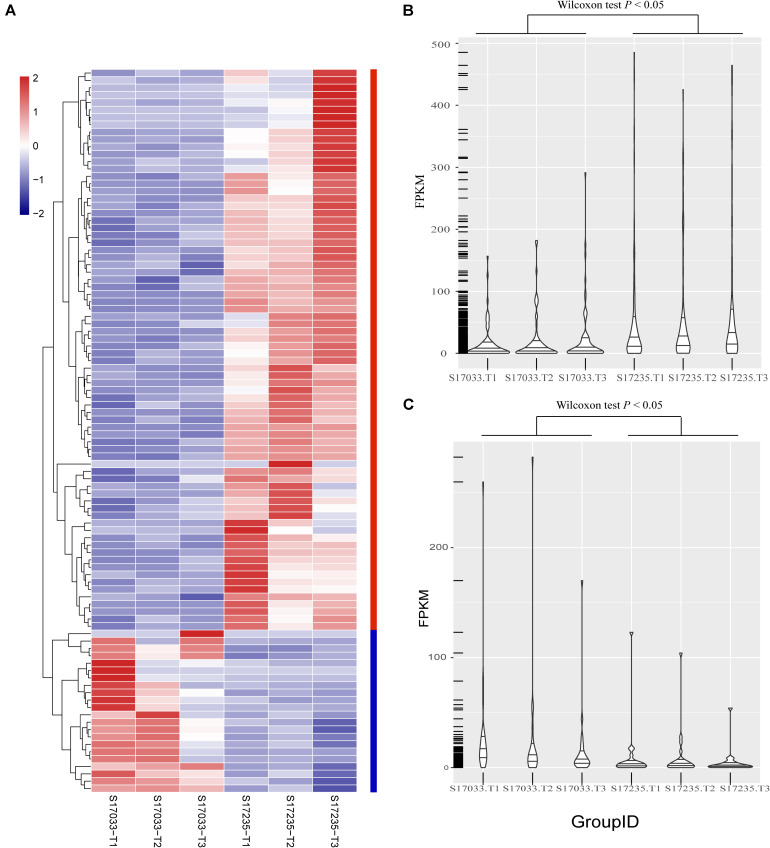
The distribution of expression levels of the 98 DEGs identified between s17033 and s17235 treatments. **(A)** The clustering analysis of the 98 DEGs, including 76 up-regulated genes and 22 down-regulated genes. **(B)** The distribution of the FPKM values of 76 up-regulated genes in s17033 and s17235, the Wilcoxon test was performed using R. **(C)** The distribution of FPKM values of 22 down-regulated genes in s17033 and s17235. The Wilcoxon test was performed using R. the order of 98 DEGs in the clustering analysis was according to the order listed in Supplementary Table S15. The labels of T1, T2, and T3 indicate the three biological replicates in the treatments of two varieties.

Secondly, DEGs between varieties were analyzed. There were 359 (33.43% up regulated) and 98 (77.55% up regulated) DEGs, including 39 and 15 novel genes, in comparison of s17033-CK VS s17235-CK and s17033-T VS s17235-T, respectively (Figure 3 and Supplementary Figure S5). A large number of genes were differently expressed between the varieties under control than under drought stress, suggesting that more DEGs were specifically involved in the seed germination in the different varieties. We focus on DEGs in comparison of s17033-T VS s17235-T. Among them, 76 and 22 DEGs were up-regulated and down-regulated, respectively. The Wilcoxon test indicated that the distribution of the FPKM values of the 76 up-regulated genes and the 22 down-regulated were significantly different (Figure 4 and Supplementary Table S15). Sixteen out of the 98 DEGs under drought stress were the same as those under control (Supplementary Figure S5), meaning that they played basic roles in seed germination regardless of variety specification. The remained 82 DEGs were considered as candidates that were responsible to the contrast drought-tolerance between s17235 and s17033 (Table 1). The 82 DEGs were assigned to 648 GO terms. In the biological process ontology, the dominant terms were “metabolic process,” “cellular process,” and “single-organism process.” The processes represented by the GO terms “cell part,” “cell,” “membrane,” and “membrane part,” accounted for the majority of the cellular components. Regarding molecular function, the dominant terms were “catalytic activity,” and “binding” (Supplementary Figure S7). The 82 DEGs were mapped to 11 KEGG pathways with the most enriched items as plant hormone signal transduction, metabolic pathways, biosynthesis of secondary metabolites, and plant-pathogen interaction (Supplementary Figure S7 and Supplementary Table S16).

**TABLE 1:** DEGs in comparison of s17033-T vs. s17235-T.

**KEGG pathway**	**Gene ID**	**Fold change (logFC)**	**NR_defination**
Galactose metabolism	GeneID:108320924	1.5	UDP-glucose 4-epimerase GEPI48
	GeneID:108330722	1.3	Probable galactinol–sucrose galactosyltransferase 2
Carbohydrate metabolism	GeneID:108321416	1.43	Thermosensitive gluconokinase
	GeneID:108331678	2.0	Pectinesterase/pectinesterase inhibitor 51
	GeneID:108332042	1.2	Pectinesterase/pectinesterase inhibitor 61
Phenylpropanoid biosynthesis	GeneID:108320083	−2.68	Mannitol dehydrogenase
Nitrogen metabolism	GeneID:108338372	1.2	Beta carbonic anhydrase 5
Lipid metabolism	GeneID:108342800	1.3	Bifunctional epoxide hydrolase 2-like
	NC_030637.1.1699	−2.2	Lipid phosphate phosphatase beta
Fatty acid elongation	GeneID:108322330	2.0	3-Ketoacyl-CoA synthase 1
Cholesterol metabolism	GeneID:108342811	−1.1	Niemann-Pick C1 protein
Acyltransferases	GeneID:108328023	1.9	Acyltransferase-like protein
Oxidative phosphorylation	GeneID:15382797	1.47	ATPase subunit 1
Energy metabolism	GeneID:108335287	−1.6	Ubiquinol oxidase 1
Amino sugar and nucleotide sugar metabolism	GeneID:108318755	4.7	Chitinase 2-like
	GeneID:108320767	−1.29	Adenylosuccinate synthetase 2
	GeneID:108329290	1.66	Proline dehydrogenase 2
Plant hormone signal transduction	GeneID:108332400	1.0	Indole-3-acetic acid-amido synthetase GH3.6-like
	GeneID:108335094	1.3	Indole-3-acetic acid-amido synthetase GH3.1
	GeneID:108334290	4.5	Auxin-induced protein 22C
	GeneID:108336351	1.52	DELLA protein GAI isoform X1
	GeneID:108320438	1.6	Ethylene-responsive transcription factor ERF084-like
	NC_030645.1.638	3.9	Ethylene-responsive transcription factor 4
	NC_030641.1.860	1.5	Ethylene-responsive transcription factor 4-like
Ubiquitin proteasome system	GeneID:108332901	1.54	F-box protein
	GeneID:108337045	1.8	F-box protein PP2-B10-like
	GeneID:108319922	1.2	F-box/kelch-repeat protein
	GeneID:108329730	−1.12	F-box/kelch-repeat protein
Transcription factor	GeneID:108331170	6.0	Transcription factor bHLH041 isoform X1
	NC_030637.1.548	1.3	Transcription factor MYB44-like
	GeneID:108331246	3.9	Transcription factor RAX3-like
	GeneID:108338278	1.1	BTB/POZ domain-containing protein
	GeneID:108346333	−2.5	NAC domain-containing protein 5-like
Transporters	GeneID:108343482	2.31	Tonoplast dicarboxylate transporter-like
	GeneID:108338861	1.5	Aquaporin TIP1-2-like
	GeneID:108344719	1.3	Organic cation/carnitine transporter 3-like
Ribosome	GeneID:108324942	1.14	50S ribosomal protein L3-2
Ribosome biogenesis	GeneID:108331859	−5.1	DEAD-box ATP-dependent RNA helicase 27-like
mRNA processing factors	GeneID:108330830	2.2	Zinc finger CCCH domain-containing protein 23-like
RNA transport	GeneID:108333126	1.2	Eukaryotic translation initiation factor 3 subunit H-like
Acylaminoacyl-peptidase	GeneID:108318938	4.5	Acylamino-acid-releasing enzyme 2-like
Mitochondrial biogenesis	GeneID:108324366	5.4	Pentatricopeptide repeat-containing protein
Autophagy	GeneID:108324651	7.4	Autophagy-related protein 9-like
Others	GeneID:108321686	6.18	Extensin-2-like
	NC_030641.1.47	6.2	Protein TIC 20-IV
	GeneID:108328075	2.5	Disease resistance RPP13-like protein 3
	GeneID:108341046	1.5	Stem-specific protein TSJT1-like
	GeneID:108334550	1.9	WAT1-related protein
	GeneID:108325033	22.50	UPF0235 protein
	GeneID:108327972	−3.7	Dirigent protein 22-like
	GeneID:108330972	−1.1	Delta(8)-fatty-acid desaturase 2-like
	GeneID:108331036	−1.8	Random slug protein 5-like
Uncharacterized or hypothetical protein	GeneID:108330014	1.1	Uncharacterized protein
	GeneID:108347396	3.11	Uncharacterized protein
	GeneID:108333186	1.5	Uncharacterized protein
	NC_030645.1.1284	2.35	Hypothetical protein
	NC_030638.1.635	1.6	Hypothetical protein
	GeneID:108325985	6.2	Hypothetical protein
	NC_030638.1.876	4.2	Hypothetical protein
	GeneID:108328410	6.0	Hypothetical protein
	NC_030645.1.1276	1.4	Hypothetical protein
	GeneID:108345321	1.1	Uncharacterized protein
	GeneID:108324359	1.53	Uncharacterized protein
	GeneID:108342424	2.2	Uncharacterized protein
	NC_030639.1.1312	1.8	Uncharacterized protein
	GeneID:108330029	1.6	Uncharacterized protein
	GeneID:108334581	2.1	Uncharacterized protein
	GeneID:108340504	1.83	Uncharacterized protein
	GeneID:108337141	1.9	Uncharacterized protein
	GeneID:108330078	1.1	Uncharacterized protein
	NC_030641.1.808	10.3	Hypothetical protein
	GeneID:108323100	9.5	Uncharacterized protein
	GeneID:108347297	6.61	Uncharacterized protein
	GeneID:108325584	2.4	Uncharacterized protein
	GeneID:108339762	1.4	Uncharacterized protein
	GeneID:108342278	1.7	Uncharacterized protein
	NC_030637.1.1874	2.7	Hypothetical protein
	GeneID:108342067	3.52	Hypothetical protein
	NC_030647.1.99	−22.5	Hypothetical protein
	GeneID:108325707	−1.6	Uncharacterized protein
	NC_030647.1.267	−2.7	Uncharacterized protein
	GeneID:108328534	−21.4	Uncharacterized protein

### qRT-PCR Validation of Differentially Expressed Genes

**FIGURE 5:**
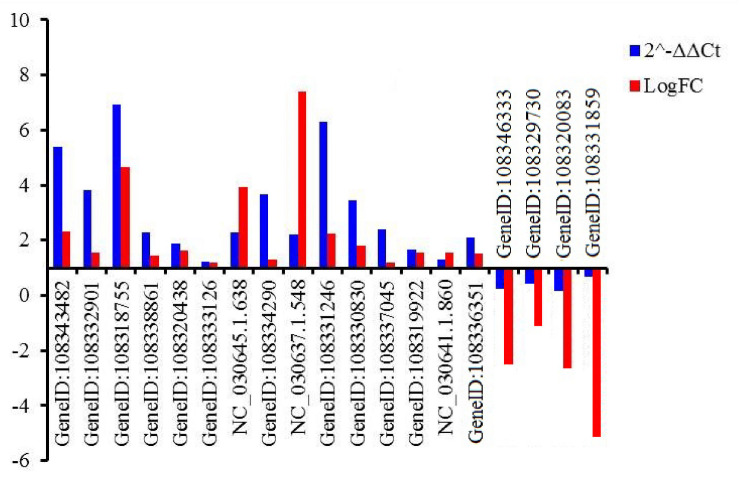
Relative expression levels of 19 genes evaluated by qRT-PCR. An actin gene of adzuki bean was used as internal control. GeneID:108343482 (TDT), GeneID:108332901 (F-box), 108318755 (CHI2L), GeneID:108338861 (TIP), GeneID:108320438 (ERF), GeneID:108333126 (EIF), GeneID:NC_030645.1.638 (ERF), GeneID: (108334290) (AIP), GeneID:NC_030637.1.548 (MYB), GeneID:108331246 (RAX3), GeneID:108330830 (ZF), GeneID:108337045 (F-box), GeneID:108319922 (KEL), GeneID:NC_030641.1.860 (ERF), GeneID:108336351 (GAI), GeneID:108346333 (NAC), GeneID: 108329730 (KEL), GeneID:108320083 (MTD), GeneID:108331859 (RH27).

Nineteen genes were randomly selected to validate the gene expression levels using qRT-PCR. Those genes encoded tonoplast dicarboxylate transporter-like (TDT, GeneID:108343482), F-box protein (GeneID:108332901, 108319922, 108337045, 108329730), chitinase 2-like protein (CHI2L, 108318755), aquaporin TIP1-2-like protein (TIP, GeneID:108338861), ethylene-responsive transcription factor (ERF, GeneID:108320438, NC_030645.1.638, NC_030641.1.860), eukaryotic translation initiation factor 3 subunit H-like protein (EIF, GeneID:108333126), auxin-induced protein 22C (AIP, GeneID:108334290), transcription factor (MYB, NC_030637.1.548; RAX3, GeneID:108331246), zinc finger CCCH domain-containing protein (ZF, GeneID:108330830), DELLA protein GAI isoform X1 (GAI, GeneID:108336351), NAC domain-containing protein (NAC, GeneID:108346333), probable mannitol dehydrogenase (MTD, GeneID:108320083), DEAD-box ATP-dependent RNA helicase 27-like (RH27, GeneID:108331859) (Supplementary Table S17). The expressed pattern of the 19 genes were consistent between q-PCR and RNA-Seq data (Figure 5), indicating that the expression levels of the identified DEGs by RNA-Seq was reliable.

## Discussion

In this study, three key findings are donated to understand the genome of adzuki bean currently. Firstly, 2,457 gene loci were newly discovered; secondly, 46,177 isoforms were identified that were absent from the Vigan 1.1 reference annotation and were defined as novel isoforms; thirdly, the length and the quality of adzuki bean transcript were significantly improved by the third generation sequencing. These findings highlighted that the combination of SMRT and NGS sequencing strategy was as an effective strategy for compensating the existing annotation of Vigan 1.1. These newly identified genes and isoforms provide valuable insight for future research on adzuki gene clones as well as functional studies of genome evolution.

NGS is limited in assembling the full-length transcripts for AS analysis. The SMRT sequencing technology overcomes these limitations where the full-length transcript is usually represented by one read. In addition, the DNA modification could be detected based on Nanopore long-read sequencing technology (Xiao et al., 2018; Liu et al., 2019). SMRT reads’s errors (mostly 1 bp insertion or deletion) could be corrected via some bioinformatic tools, such as LoRDEC and MECAT, and had minimal effects on the AS analysis of sequenced genomes (Li et al., 2017; Xiao et al., 2017). As the proof, larger numbers of unique isoforms and the greater length of transcription were specifically generated by SMRT derived reads (Figure 1).

AS is crucial in regulation of plant development and stress responses (Thatcher et al., 2016). By analysis of the AS dynamics during the germinating seeds of two contrast drought-tolerant adzuki bean, the drought-tolerant variety s17235 showed greater change in four AS event than drought sensitive variety s17033 between control and drought stress. On the other hand, the control group had the bigger number in each AS types than stress group in two varieties, implying that AS occurred and even generated more events in plant normal growth. The number of AS events reduced slightly or remained roughly the same under drought stress compared to the control, suggesting that most of the AS activity is necessary to perform their physiological functions for plant normal growth and may turn off the expression of genes that are not essential for life. It was demonstrated that stress-associated genes were liable to AS, and AS largely targeted ABA pathway to regulate stress responses (Laloum et al., 2017). In this study, some genes related to drought stress were detected with specific AS event between two varieties under drought stress, such as protein phosphatase 2C (PP2C) (108345311), heat shock 70 kDa protein (HSP) (108320180), late embryogenesis abundant (LEA) protein (108324617) (Table 1), indicating that AS might regulate the seed germination by targeting those genes. PP2C phosphatases are key regulator factor in ABA pathway, which directly regulate SnRK2 kinases (Mehta et al., 2017). LEA is regulated by the ABA signal pathway genes ABI3, ABI4, ABI5 and DOG1 in the process of seed maturation (Leprince et al., 2016). HSPs are involved in protein folding, assembly, translocation, and degradation as chaperones, which keep cells not injured and facilitate recovery and survival when normal growth conditions were returned (Mehta et al., 2017). Homologs of these genes were identified with drought stress response in tobacco, *Chenopodium quinoa*, *S. miltiorrhiza* (Vranová et al., 2000; Wang H. et al., 2017; Liu et al., 2018).

Eighteen-two DEGs were identified between the two varieties under drought stress (the comparison of s17033-T vs. s17235-T, Table 1), which might be responsive for differential drought tolerant. Seed germination begins with imbibition and ends with radicle emergence. Quantities of gene expression changed in this physiological process, including those involved in carbohydrate, amino acids and cell wall metabolism, transcription and translation, phytohormones (Han and Yang, 2015). Among the 82 DEGs, 17 DEGs are involved in metabolism and five genes (GeneID: 108324942, 108331859, 108330830, 108333126, 108318938) participate in transcription or translation, suggesting that the energy providing or efficiency of gene transcription or translation affect the seed germination of the two varieties. Meanwhile, DEGs participate in plant hormone signal transduction, such as auxin indole-3-acetic acid (IAA) (GeneID: 108332400, 108335094, 108334290), Gibberellin (GA) (GeneID: 108336351), and ethylene (ET) (GeneID: 108320438, NC_030645.1.638, NC_030641.1.860). It was documented that ABA and GA were two primary hormones that repressed and promoted seed germination, whereas auxin and ET retained and brought seed dormancy by regulating ABA signaling, respectively (Shu et al., 2016). In this study, the seven DEGs were all up-regulated in drought tolerant variety (Table 1), implying that the mechanism of regulating seed germination involved in hormone signaling is complicated.

The ubiquitin proteasome system plays a key regulatory roles in many biological processes of eukaryotes (Lechner et al., 2006). F-box protein is one important component of ubiquitin proteasome system which functions in recruiting the target substrate. F-BOX protein were reported to regulate seed germination in *Arabidopsis* (He et al., 2016; Majee et al., 2018). TFs are critical regulatory factors for developmental processes and stress responses in plant. TFs bHLH, MYB, NAC participated in regulating seed germination in rice, wheat, and *Arabidopsis*, respectively (Chen et al., 2018; Zhao et al., 2018; Guan et al., 2019). Four DEGs encoding ubiquitin proteasome system proteins (GeneID: 108332901, 108337045, 108319922, 108329730), and five TFs (GeneID: 108331170, NC_030637.1.548, 108331246, 108338278, 108346333) were identified, indicating they might regulate seed germination in response to drought stress in adzuki bean.

Aquaporins, the membrane channels, transport water and small neutral molecules across biological membranes of living organisms (Maurel et al., 2015). The aquaporin TIP1-2 is one of the tonoplast intrinsic proteins (TIPs) subfamilies, which is permeable to urea, ammonia, and hydrogen peroxide in *Arabidopsis* (Schüssler et al., 2008). Autophagy is an intercellular degradation/recycling system. In plant, autophagy is involved in recycling nutrients and combating with biotic and abiotic stresses (Chen et al., 2017). Recently, a dehydrin MtCAS31 (cold acclimation-specific 31) of *Medicago truncatula* was showed to participate in the autophagic degradation pathway by positively regulating drought response. It can interact with the aquaporin MtPIP2;7 protein MtCAS31, which is a negative regulator of drought response. MtCAS31 promoted degradation of MtPIP2;7 in the autophagic pathway so that water loss was reduced and drought tolerance was improved under drought stress (Li et al., 2019). In this study, one TIP1-2-like gene (GeneID: 108338861) and one autophagy-related gene (GeneID: 108324651) were significantly up-regulated in drought tolerant variety under stress (Table 1), suggesting that they play crucial roles in regulation seed germination to acclimatize to drought response.

## Conclusion

In this study, a large number of newly loci and isoforms were discovered by means of the large-scale transcriptome sequencing in adzuki bean, which supplement the insufficient transcriptomic and genomic data of adzuki bean. On the other hand, numerous AS events and DEGs were characterized to be associated with drought response during seed germination in adzuki bean. The genes undergoing AS events or transcription changes are potential candidate in regulation of seed germination in response to drought stress. The results are found for further investigation of the adaptation to drought in adzuki bean. Additionally, the transcriptome sequences can serve for improvement programs by genomics-assisted genetics, and facilitate to understand and manipulate the biochemical pathways for developing drought-tolerant crop plants.

## Materials and Methods

### Plant Materials

The seeds of drought-tolerant and drought-sensitive variety s17235 (XD213) and s17033 (XD032) were treated with mannitol solution or distilled water as control following the method described by Zhu et al. (2019). To comprehensively investigate the adzuki bean transcriptome during seed germination in response to drought, the seeds of s17235 and s17033 germinating in mannitol solution or deionized water for 24 h were selected for NGS and SMART sequencing. For NGS, 12 libraries were constructed, that is, s17235 and s17033 germinating in mannitol solution, which was referred to s17235-T1, s17235-T2, s17235-T3, s17033-T1, s17033-T2, s17033-T3, respectively. s17235 and s17033 germinating in deionized water were as controls, which was referred to s17235-CK1, s17235-CK2, s17235-CK3, s17033-CK1, s17033-CK2, s17033-CK3, respectively. For SMART sequencing, all the RNAs of six samples of s17235 or s17033 were equally mixed to form two variety-specific samples, respectively. Then the two variety-specific samples were used to construct three libraries with three size ranges, 1–2, 2–3, and > 3 kb, and were used for single-molecule real-time sequencing using the PacBio RS II platform. The seeds were collected and immediately frozen with liquid nitrogen, stocked in −80°C. Total RNA were isolated from the germinating seeds of s17235 and s17033 using a commercial Kit (Takara Biotechnology, Dalian, China). The purified RNA was dissolved with RNase-free water, and removed genomic DNA contamination using TURBO DNase I (Promega, Beijing, China). The integrity of the RNA was determined with the Agilent 2100 Bioanalyzer (Agilent Technologies, Palo Alto, CA, United States). When RIN value was ≥ 8, the total RNA samples were used for constructing the cDNA libraries in PacBio or HiSeq sequencing.

### PacBio Library Construction and Sequencing

Total RNA (2 μg) was reversely transcribed into cDNA with SMARTER PCR cDNA Synthesis Kit with optimization for prepare of high-quality, full-length cDNAs (Takara Biotechnology, Dalian, China), followed by size fractionation using the BluePippin Size Selection System (Sage Science, Beverly, MA, United States). Each SMRT bell library was constructed using 1–2 μg size-selected cDNA with the Pacific Biosciences DNA Template Prep Kit 2.0. The binding of SMRT bell templates to polymerases was conducted using the DNA/Polymerase Binding Kit P4 and v3 primers. Sequencing was conducted on the Pacific Bioscience sequel platform using C3 reagents with 120 min movies.

### Illumina Library Construction and Sequencing

The cDNA library was constructed with NEBNext Ultra RNA Library Prep Kit for Illumina (NEB, E7530) and NEBNext Multiplex Oligos for Illumina (NEB, E7500) according to the manufacturer’s instructions. Briefly, approximately 250∼300 bp RNA inserts were fragmented from the enriched mRNA. Then, the first-strand cDNA and second cDNA were synthesized from them. End-repair/dA-tail and adaptor ligation were performed for the double-stranded cDNA. Agencourt AMPure XP beads (Beckman Coulter, Inc.) were used to isolate the suitable fragments, and enriched by PCR amplification. Finally, sequencing of the constructed cDNA libraries were performed on an Illumina HiSeqX sequencing platform.

### Subread Processing and Error Correction

P_Fetch and P_Filter function (parameters: minSubReadLength = 50 and readScore = 0.65) were used to obtain the effective subreads in the SMRT Analysis Software v2.3 Suite^2^. The FL transcript sequence was obtained using ToFU pipeline (Gordon et al., 2015) (Figure 1A). In Brief, circular consensus (CCS) read was produced from the P_CCS module with the parameter MinFullPasses = 1 and MinPredictedAccuracy = 0.8. After examining for poly(A) signal, 5′ and 3′ adaptors, only the CCS with all three signals was considered a FL non-chimetric (FLNC) read (Minoche et al., 2015). To improve consensus accuracy, we used an isoform-level clustering algorithm, namely, iterative clustering for error correction (ICE), and polished FL consensus sequences from ICE using Quiver. Additional nucleotide errors in FLNC reads were corrected by the Illumina RNA-seq data with the software Proovread (Hackl et al., 2014) using the parameter coverage of 127. The uncorrected and corrected FLNC sequences were aligned to adzuki bean genome sequence through GMAP, respectively (Wu and Watanabe, 2005). The untrimmed sequence was considered as the result of error correction.

### Mapping of PacBio Data

The error rectified FLNC reads were mapped to the reference genome sequence using GMAP26 with the options-no-chimeras-n20 (Figure 1B). The best mapped locus was selected for each FLNC read based on both identity and coverage values. The loci and isoform were annotate with the high percent of identity (PID) aligned FLNC reads. The same loci transcript meet the requirements that 20% overlap for two sequences and at least one exon overlapping to more than 20%. For isoform, single-exon sequence with overlap was determined as the same isoform. The same isoform was considered when the multiple-exon sequences have all identical splicing sites. The redundant and false positive gene structure was removed as follows: (i) the missing 5′ end was removed; the sequence structure was a subset of other sequences (sequence structure refers to the ordered sets of all remaining cleavage sites, excluding the initiation and termination sites); the 5′ last exon spanning the intron region was determined, and the sequence was retained when it spanned the intron region; (ii) region PID < 99: at least two PacBio sequences were kept for each transcript model; otherwise, the junction of second-generation sequencing annotated or supported all junctions of this sequence and (iii) the longest one was kept when the structure of two sequences was the same.

### Novel Isoform

The gene structure annotation results were compared with those of reference annotation to determine the new gene following these criteria: (1) less than 20% of the annotated gene site showed no overlap or overlapped, or (2) the gene overlap was more than 20%, but the gene direction was not consistent. The criteria used for a single transcript to identify novel isoform were as follows: (i) the isoform existed one or more new splicing sites (ii) The annotated gene transcripts and the Isoform obtained by sequencing are not single exons at the same time (Figure 1D).

### Alternative Splicing Classification

AS profile software (Florea et al., 2013) was used to classify and count the isoform variable splicing events. The AStalavista was used to identify AS of transcripts at FPKM ≥ 0.01 threshold in any biological repeat. Four major types of AS events, namely IR (intron retention), ES (exon skipping), AA (alternative 3′ splice site), and AD (alternative 5′ splice site) were characterized.

### DEGs and Isoforms Identifying and Functional Categorizing

Comparison of gene expression was achieved by the edgeR package (Robinson et al., 2010). The *t*-test was used to judge the statistical significance of expression difference, and the threshold of *P*-value was determined with the FDR in multiple testing. In this study, DEGs were filtered with RPKM ≥ 0.1, |log2 fold change| ≥ 2, and FDR ≤ 0.05 or FDR ≤ 0.01 in each pairwise comparison between varieties and treatment. GO annotations was assigned by use of Blast2GO (Conesa et al., 2005), and assigned metabolic pathway annotations by blast against the KEGG database. Hyper-geometric tests were used to both GO and KEGG pathway enrichment analyses for the DEGs with the whole seed transcriptome as the background.

### qRT-PCR Assay

qRT-PCR was conducted according to Sha et al. (2016) with minor modification. Briefly, total RNAs were extracted by Trizol reagent (Tiangen, China), and first-strand cDNA was prepared from 5 μg of total RNA using the First-Strand cDNA Synthesis Kit (Promega, United States). Primers were designed for 19 were adversity stress-related genes as listed in Supplementary Table S17. The reactions were performed on a BIO-RAD CFX Connect Real Time System (BIO-RAD, United States) following the manufacturer’s instructions. 20 μl volume for each reaction mixture. It comprised 10 μl of SYBR Premix Ex Taq (Tiangen, China), 0.5 μl of each primer (10 μM), 1 μl of cDNA template, and 8 μl of RNase-free water. The reactions for each gene were conducted in triplicate with the thermal cycling conditions as follows: 95°C for 15 m, followed by 40 cycles of 95°C for 10 s, 60°C for 20 s and 72°C for 30 s,the last stage is 95°C for 10 m, 60°C for 5 s and 95°C. The primer specificity was confirmed by melting curve analysis. The relative expression levels of the tested genes were calculated using the 2^–Δ^ ^Δ^ ^*Ct*^ method with normalization to that of the reference genes.

## Data Availability Statement

The datasets generated or analyzed for this study can be found in the SRA: https://dataview.ncbi.nlm.nih.gov/object/PRJNA577173?reviewer=3i3tj5uakhrcboc4clovhpqk0n.

## Author Contributions

ZZ conducted AS analysis. HC performed DEGs analysis. KX performed qPCR analysis. CL and LL screened the drought tolerance of seed and prepared the germinating seeds. LJL and XH performed the data analysis. AS and ZW designed the experiments. ZZ, HC, and KX wrote the manuscript. CJ analyzed data and critically revised the work. All authors read and approved the final manuscript.

## Conflict of Interest

The authors declare that the research was conducted in the absence of any commercial or financial relationships that could be construed as a potential conflict of interest.

## Footnotes

1 ftp://ftp.ncbi.nlm.nih.gov/genomes/all/GCF/001/190/045/GCF_001190045.1_Vigan1.1/

2 http://www.pacb.com/devnet/

## Supplementary Material

FIGURE S1 Schematic of the seven groups of PacBio isoforms. **(a)** Isoforms have been annotated in the IWGSC RefSeq v1.0. **(b)** Novel isoforms that contain multiple exons and have different splice sites and introns compared with annotated isoforms in the IWGSC RefSeq v1.0. **(c)** Novel isoforms that contain multiple exons sequentially shared the introns of the annotated isoforms in the IWGSC RefSeq v1.0. **(d)** Novel isoforms that contain multiple exons sequentially contained the introns of the annotated isoforms in the IWGSC RefSeq v1.0. **(e)** Novel isoforms that contained a single exon and the annotated isoforms that contained multiple exons, or vice versa. **(f)** Novel isoforms derived from novel gene loci. **(g)** Novel isoforms that were non-overlapping with known isoforms in the same gene loci.

FIGURE S2 The enrichment analysis of KEGG pathway for the genes with IR specifically at four comparison. The area of each colored circle is proportional to the number of genes involved in each pathway, the color indicates the Rich Factor, and the x-axis is the *q*-value.

FIGURE S3 GO and KEGG analysis for the 152 DSGs identified in both varieties with drought stress.

FIGURE S4 Correlation analysis of the RPKM (reads per kilobase per million mapped reads) values of all 12 samples. The average coefficient for the three replicates at the 13033-CK, 17033-T, 17235-CK, and 17235-T was 0.9583, 0.9076, 0.8923, and 0.9346, respectively.

FIGURE S5 Comparison of DEGs among the four comparison groups in varieties and treatment.

FIGURE S6 GO and KEGG analysis for the 2195 DEGs identified in both varieties.

FIGURE S7 GO and KEGG analysis for the 82 DEGs identified in both varieties with drought stress.

TABLE S1 General properties of the reads produced by Illumina sequencing and PacBio sequencing.

TABLE S2 The alignment of FLNC reads with the Vigan 1.1.

TABLE S3 The gene and isoform annotations of the Adzuki bean Vigan 1.1 and PacBio data.

TABLE S4 The genes and isoforms identified in the PacBio data.

TABLE S5 The BLAST hits of the newly discovered transcripts among the public databases. NR (NCBI’s non-redundant protein sequences), GO (Gene Ontology), KO (KEGG ORTHOLOG), KOG (euKaryotic Ortholog Group), Swiss-Prot (Swiss-Prot Protein Sequence Database).

TABLE S6 Statistics of alternative splicing events for each of the four samples.

TABLE S7 Enriched GO terms in the list of genes undergoing IR in four comparison groups.

TABLE S8 The list of DSGs between 17033-T and 17235-T. The genes whose names start with “NC” were newly discovered in the PacBio data.

TABLE S9 KEGG pathway enrichment analysis for the DSGs in s17033-T-vs-s17235-T.

TABLE S10 The 409 fusion transcripts that map to two or more loci in the genome.

TABLE S11 The statistics of the 133 fusion genes supported by transcripts.

TABLE S12 Putative genes encoding transcription factor involved in drought stress response identified in the seed transcriptome of Adzuki bean.

TABLE S13 List of the highly expressed genes (RPKM ≥ 1000) at four samples of Adzuki bean seeds.

TABLE S14 The list of DEGs. “1” represents the gene is differentially expressed gene (DEG) in the comparison samples. The genes whose names start with “N” were newly discovered in the PacBio data.

TABLE S15 The expression levels of the 98 DEGs identified between s17033 and s17235 treatments.

TABLE S16 KEGG pathways represented with 98 DEGs under drought-stress between s17235 and s17033 of Adzuki bean seeds.

TABLE S17 Primers for 19 genes for qRT-PCR analyses.
