# Supplementary material for: Characterization of Drought-Responsive Transcriptome During Seed Germination in Adzuki Bean (Vigna angularis L.) by PacBio SMRT and Illumina Sequencing
Source: Front Genet. 2020 Aug 31;11:996. doi: 10.3389/fgene.2020.00996 (PMC7489039; doi:10.3389/fgene.2020.00996)
Supplement: FIGURE S1 — Schematic of the seven groups of PacBio isoforms. (a) Isoforms have been annotated in the IWGSC RefSeq v1.0. (b) Novel isoforms that contain multiple exons and have different splice sites and introns compared with annotated isoforms in the IWGSC RefSeq v1.0. (c) Novel isoforms that contain multiple exons sequentially shared the introns of the annotated isoforms in the IWGSC RefSeq v1.0. (d) Novel isoforms that contain multiple exons sequentially contained the introns of the annotated isoforms in the IWGSC RefSeq v1.0. (e) Novel isoforms that contained a single exon and the annotated isoforms that contained multiple exons, or vice versa. (f) Novel isoforms derived from novel gene loci. (g) Novel isoforms that were non-overlapping with known isoforms in the same gene loci. [file Data_Sheet_1.zip › Supplementary figures and tables/Supplementary figure 1-7.docx]

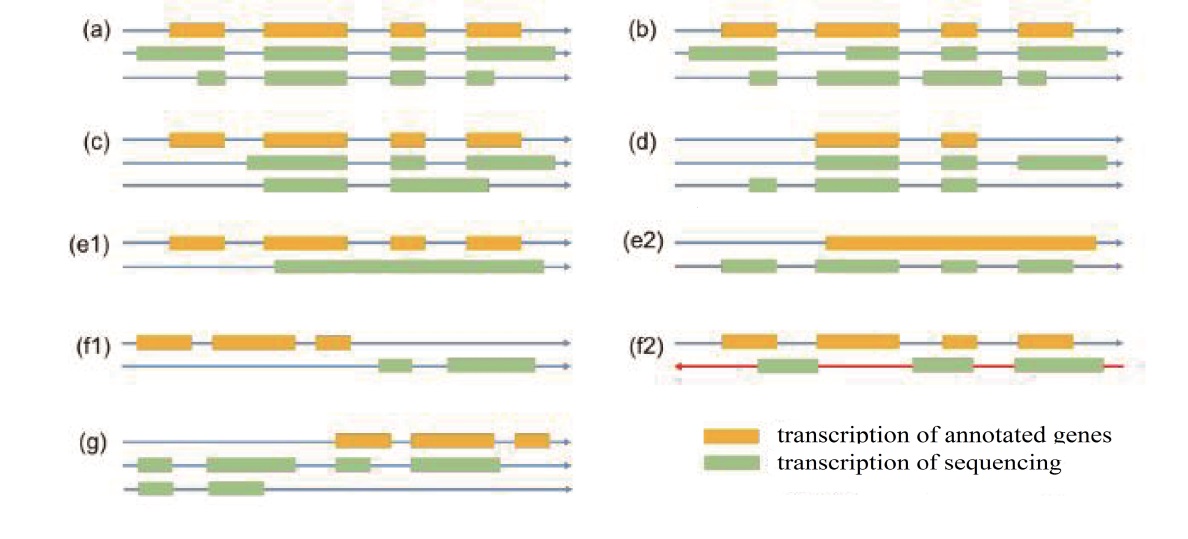


Supplementary Figure S1. Schematic of the seven groups of PacBio isoforms.(a) Isoforms have been annotated in the IWGSC RefSeq v1.0.(b) Novel isoforms that contain multiple exons and have different splice sites and introns compared with annotated isoforms in the IWGSC RefSeq v1.0. (c) Novel isoforms that contain multiple exons sequentially shared the introns of the annotated isoforms in the IWGSC RefSeq v1.0. (d) Novel isoforms that contain multiple exons sequentially contained the introns of the annotated isoforms in the IWGSC RefSeq v1.0. (e) Novel isoforms that contained a single exon and the annotated isoforms that contained multiple exons, or vice versa. (f) Novel isoforms derived from novel gene loci. (g) Novel isoforms that were non-overlapping with known isoforms in the same gene loci.


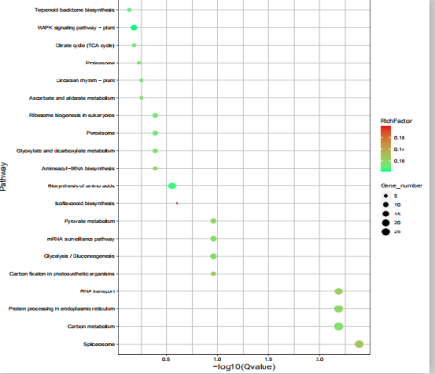

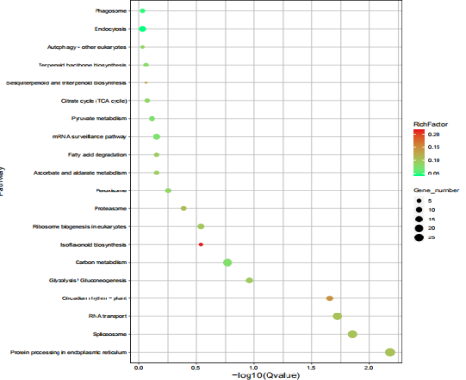

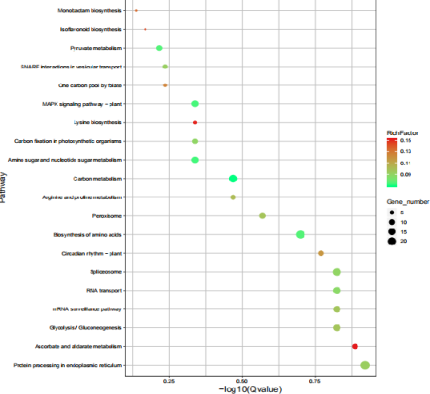

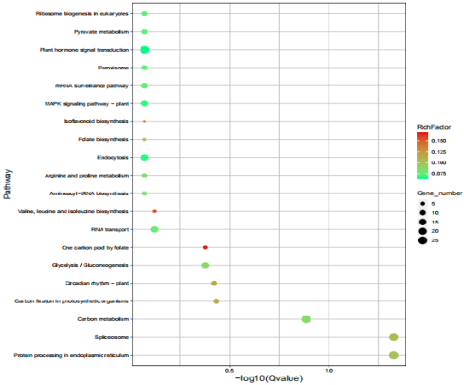


Supplementary Figure S2. The enrichment analysis of KEGG pathway for the genes with IR specifically at four comparition.The area of each colored circle is proportional to the number of genes involved in each pathway, the color indicates the Rich Factor, and the x-axis is the q-value.


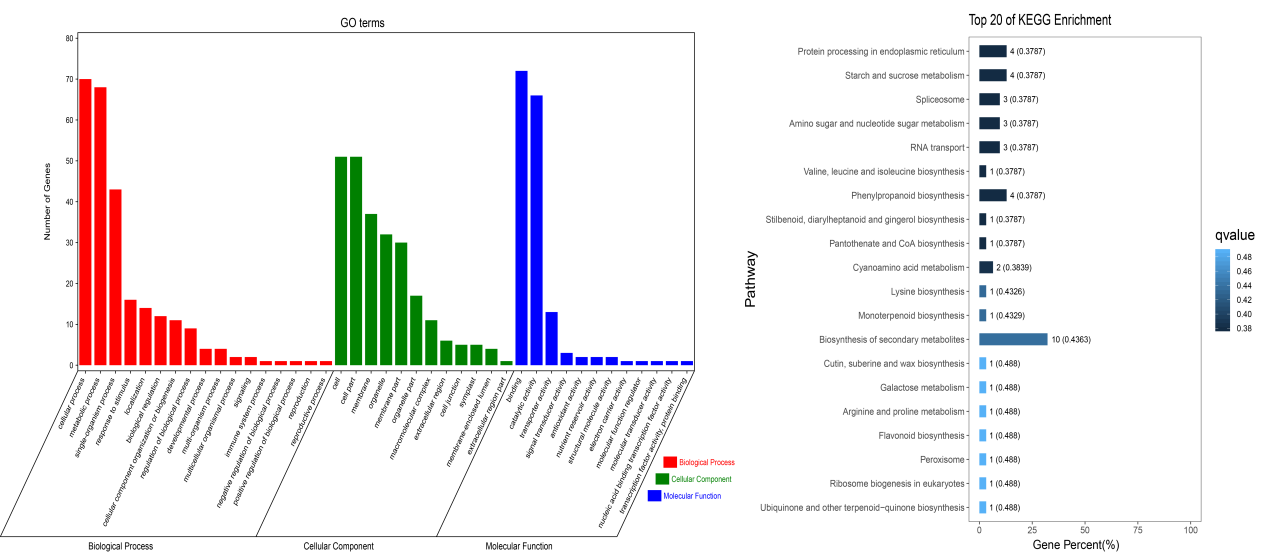


Supplementary Figure S3. GO and KEGG analysis for the 152 DSGs identified in both varieties with drought stress


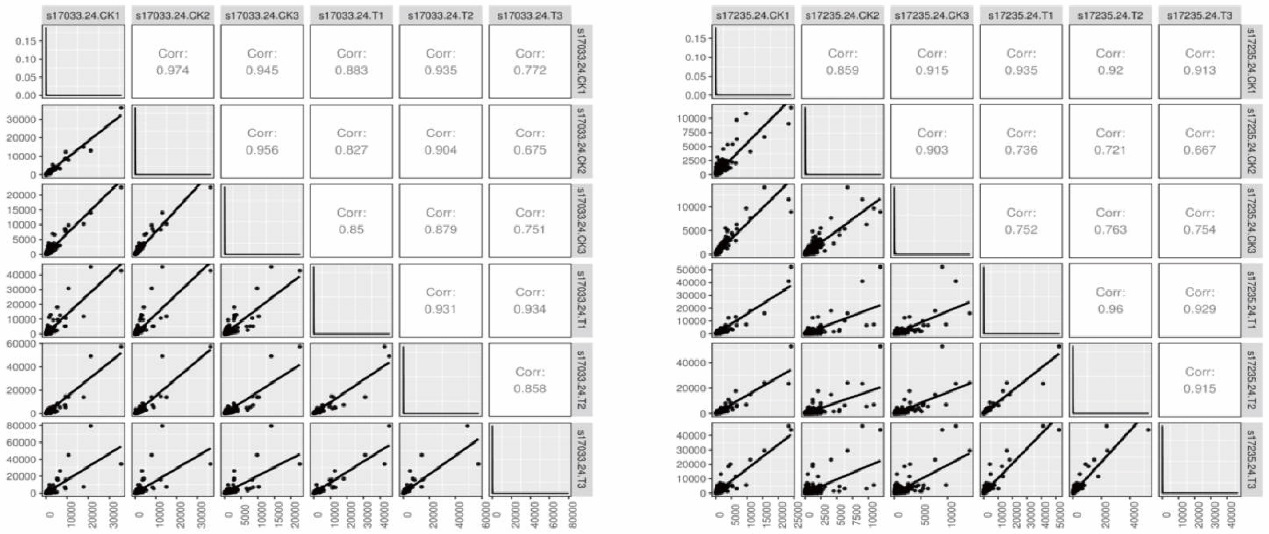


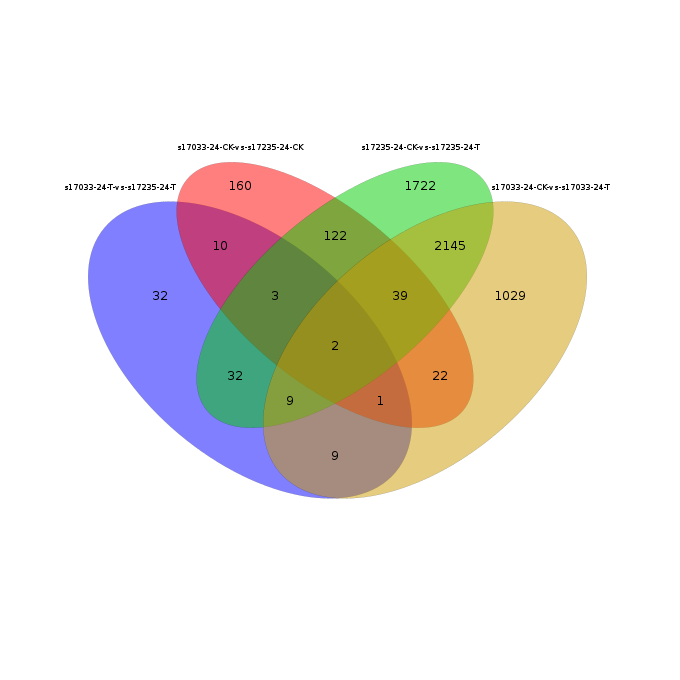
**Supplementary Figure S4**. Correlation analysis of the RPKM (reads per kilobase per million mapped reads) values of all twelve samples. The average coefficient for the three replicates at the 13033-CK, 17033-T, 17235-CK and 17235-T was 0.9583, 0.9076, 0.8923 and 0.9346, respectively

Supplementary Figure S5. Comparison of DEGs among the four comparison groups in varieties and treatment.


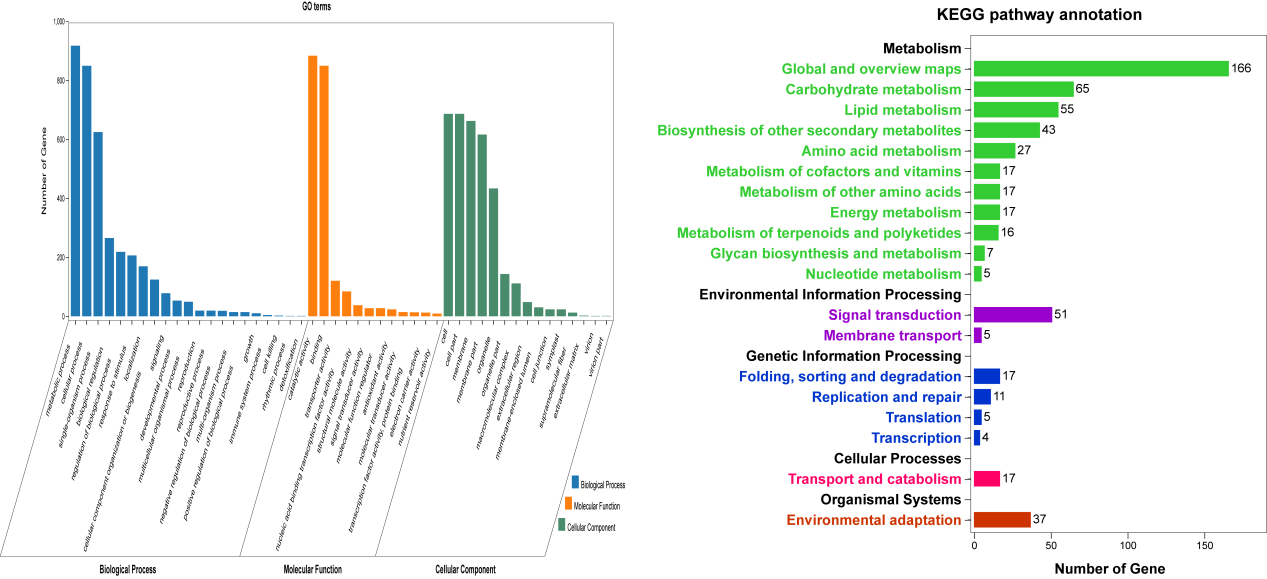


Supplementary Figure S6. GO and KEGG analysis for the 2195 DEGs identified in both varieties


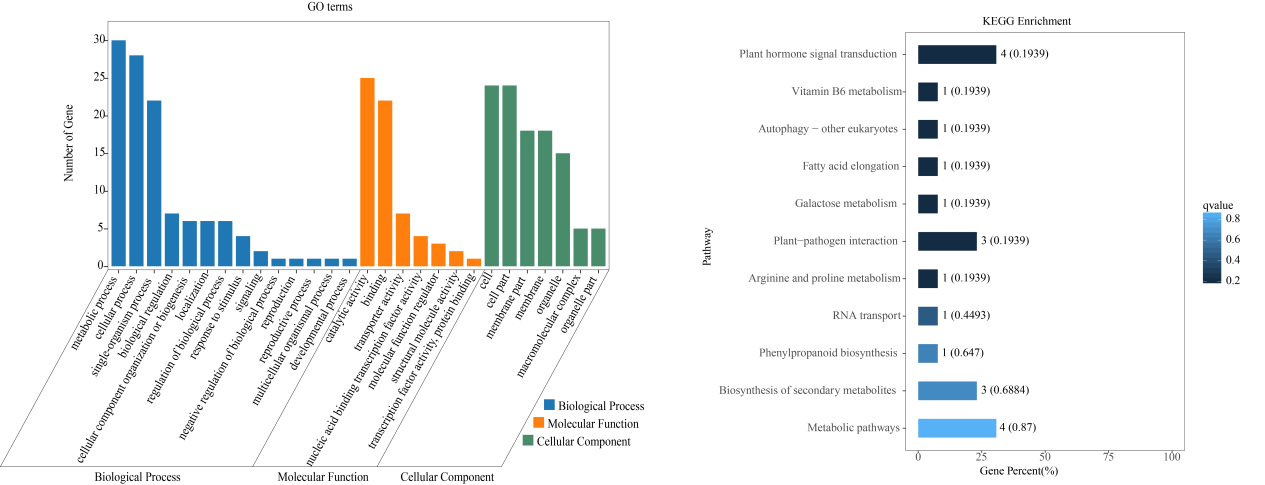


Supplementary Figure S7. GO and KEGG analysis for the 82 DEGs identified in both varieties with drought stress
